# Preprandial food-dependent exercise-induced anaphylaxis to banana

**DOI:** 10.5415/apallergy.0000000000000113

**Published:** 2023-10-09

**Authors:** Thatchai Kampitak

**Affiliations:** 1 Allergy Unit, Department of Medicine, Samitivej Sukhumvit Hospital, Bangkok, Thailand

**Keywords:** Anaphylaxis, banana, exercise-induced, FDEIA, food-dependent, preprandial

## Abstract

Food-dependent exercise-induced anaphylaxis is a disorder in which a reaction develops only in association with physical exertion that generally takes place postprandially. The reaction that occurs following food intake after exercise is uncommon. Banana is an infrequent cause of anaphylaxis, which has been previously reported in combination with postprandial exercise in only 1 patient. A probable case of preprandial food-dependent exercise-induced anaphylaxis to banana is described herein together with a brief review of recent related literature.

## 1. Introduction

Food-dependent exercise-induced anaphylaxis (FDEIA) is a rare disorder in which an anaphylactic reaction develops exclusively when physical activity occurs, usually within a few hours, following food ingestion in most cases [1, 2]. FDEIA, in which anaphylaxis occurs after eating postexercise has been reported in only a few patients previously [3, 4]. A probable case of preprandial FDEIA to banana is described herein.

## 2. Case report

A 25-year-old man developed abdominal cramps, watery diarrhea, diffuse urticaria with eyelid and lip swelling, rhinorrhea, nasal congestion, and ocular redness with pruritus almost immediately after eating 2 bananas. He then presented to the emergency room after experiencing minimal improvement with an oral antihistamine. On examination, he had no respiratory distress or cardiovascular compromise. Generalized urticaria and angioedema involving eyelids and lips were visible. Despite being treated with chlorpheniramine, dexamethasone, and pantoprazole injections, he developed another severe bout of crampy abdominal pain, vomiting, watery diarrhea, and flushing. Adrenaline was administered intramuscularly and resulted in rapid resolution of his symptoms.

He was previously healthy and denied concurrent illness or intake of drugs (including nonsteroidal anti-inflammatory drugs [NSAID]s) and alcohol. He was not atopic and had no previous history of drug, food, or latex allergies. He had 2 bananas about 2 hours after exercising in a gym with intensity and duration similar to his routine. Exercise and bananas were each tolerated independently. He had eaten bananas both before and after exercise without incident but not with more than 1 banana previously.

Serum tryptase levels at a few hours and 3 days after the reactions were 10.8 and 1.37 µg/L, respectively. Skin testing on fresh banana showed a positive reaction (wheal diameter 3 mm, histamine 3 mm) while radioallergosorbent test for specific immunoglobulin E (IgE) antibodies to banana was positive at the level of 0.46 KU/L (class 1). An exercise challenge test with banana was not carried out due to the patient concern of a potential reaction. He was diagnosed with FDEIA to banana and prescribed an epinephrine autoinjector. He continued regular exercising in a gym but not in association with banana ingestion although, eating banana independently of the exercise was later tolerated.

## 3. Discussion

FDEIA is characterized by anaphylaxis that occurs as a result of the combination of exercise and food intake when the culprit food is tolerated in the absence of exercise and vice versa [1]. It has been reported worldwide and most commonly affects adolescents and young adults with a prevalence of approximately 0.02% although the exact number might probably be higher due to underdiagnosis [1]. It has a slight male predominance (55%) with the median age at onset of 21 years (range 4–82 years) [5, 6]. Contrary to its term, not all patients diagnosed with FDEIA present with anaphylaxis. According to the recent meta-analysis of 722 patients, 3 different phenotypes were identified including anaphylaxis with wheals and/or angioedema (80%), wheals, angioedema, or both without anaphylaxis (17%), and anaphylaxis without wheals or angioedema (4%) [5]. However, the clinical significance of differentiating the phenotype for FDEIA remains to be further explored.

FDEIA can be classified into 2 types based on the culprit food including specific FDEIA (1 or multiple specific foods) and nonspecific FDEIA (any food) [2, 5]. The most commonly implicated foods include wheat (66%), vegetables (9%), seafood (9%), legumes (7%), and fruits (6%) [5]. The causative foods can be varied among regions in which wheat, other grains, and nuts are common in Western populations while wheat and shellfish are prevalent in Asian countries [1]. Banana is an infrequent, but increasingly recognized, cause of anaphylaxis and has been previously reported in association with FDEIA in only 1 case [7, 8]. Banana allergy affects about 0.04% to 1.2% of the general population and is more prevalent among asthmatic and atopic dermatitis individuals [9]. It can be associated with pollen fruit allergy syndrome, latex-fruit syndrome, Kounis syndrome, and anaphylaxis [8–10]. There are at least 6 major banana allergens including Mus a1 (profilin), Mus a2 (class I chitinase), Mus a3 (nonspecific lipid transfer protein), Mus a4 (thaumatin-like protein), Mus a5 (β-1,3-glucanase), and Mus a6 (ascorbate peroxidase) of which Mus a1 and Mus a2 are responsible for pollen fruit allergy syndrome and latex-fruit syndrome, respectively, although profilin susceptibility accounts for most reactions due to banana allergy [8, 9].

FDEIA can be associated with a variety of physical exercise including running, walking, playing sports, and dancing [5]. Low levels of exertion may be sufficient to trigger symptoms in some patients, particularly the elderly [1]. Most patients develop reactions within 2 hours after eating (median 110 minutes, range 15 minutes–7 hours) and 1 hour after exercise (median 30 minutes, range 5 minutes–5 hours) while the elapsed time between meal and exercise ranges from 5 minutes to 6 hours (median 1 hour) [5]. FDEIA, in which a reaction occurs after exercise followed by food ingestion is uncommon and has been previously described in only a few cases, including a 39-year-old woman who developed anaphylaxis following celery ingestion within 2 hours after playing tennis and a 27-year-old woman whose anaphylaxis occurred after eating raspberries, peaches, and cranberries shortly after exercise [3, 4].

The exact mechanism responsible for FDEIA remains unclear. It was postulated that exercise can induce anaphylaxis in food-sensitized patients by either accelerating food absorption or lowering the anaphylaxis threshold level [1]. However, it was demonstrated that patients with wheat-dependent exercise-induced anaphylaxis could elicit symptoms after being challenged with high gluten doses with or without aspirin and alcohol in the absence of exercise [11]. Therefore, it may be assumed that FDEIA is a variant form of food allergy with high threshold levels that could possibly be lowered by cofactors, including exercise, NSAIDs, alcohol, menstruation, infections, physical illness or stress, and extreme environment, and the term cofactor-dependent food allergy or augmentation factor-triggered food allergy might be more appropriate [11, 12]. In addition, it might be possible that in the existence of multiple triggering factors, such as large amount of food, strenuous exercise, and extreme weather, FDEIA may develop regardless of the sequence [4].

FDEIA remains a clinical diagnosis which is largely based on comprehensive history taking for identifying the culprit food [1]. Skin testing, with a commercial extract or fresh food, or in vitro testing may be helpful to confirm the diagnosis, particularly specific IgE to omega-5-gliadin for wheat-dependent exercise-induced anaphylaxis. A provocation test is the gold standard for diagnosing FDEIA although it may show a false-negative result due to intraindividual variability [2]. Avoidance of the culprit food in combination with exercise as well as identification of other augmenting factors is the mainstay management since most patients experience no more reactions when they no longer eat the culprit food with exercise while the efficacy of the prophylactic treatment for FDEIA remains uncertain [1, 2].

In conclusion, although uncommon, banana should be recognized as a food that may be responsible for FDEIA even though it is eaten in the postexercise period.

## Acknowledgement

None.

## Conflicts of interest

The authors have no financial conflicts of interest.

## Author contributions

Thatchai Kampitak: clinical treatment, conception and design, data acquisition, drafting, review, and final approval of the article.

## Patient consent

An informed consent was obtained from the patient.
